# All-optical computing towards 100-GHz clock rates

**DOI:** 10.1038/s41377-026-02314-5

**Published:** 2026-07-17

**Authors:** Gordon H. Y. Li, Midya Parto, Jinhao Ge, Qing-Xin Ji, Maodong Gao, Yan Yu, James Williams, Robert M. Gray, Christian R. Leefmans, Nicolas Englebert, Kerry J. Vahala, Alireza Marandi

**Affiliations:** 1 Department of Applied Physics, California Institute of Technology, Pasadena, 91125 CA USA; 2 Department of Electrical Engineering, California Institute of Technology, Pasadena, 91125 CA USA; 3 Physics and Informatics Laboratories, NTT Research, Inc., Sunnyvale, 94085 CA USA; 4 CREOL, The College of Optics and Photonics, University of Central Florida, Orlando, FL USA; 5 T. J. Watson Laboratory of Applied Physics, California Institute of Technology, Pasadena, 91125 CA USA

**Keywords:** Nonlinear optics, Ultrafast photonics

## Abstract

A computer’s clock rate ultimately determines the minimum time between sequential operations or instructions. Despite exponential advances in electronic computer performance due to Moore’s Law and increasingly parallel system architectures, clock rates of conventional processors have remained stagnant at ~5 GHz for nearly two decades. This creates a significant challenge for applications requiring real-time processing or control of ultrafast information systems. Here, we break this barrier by proposing and experimentally demonstrating computing based on an all-optical recurrent neural network leveraging the ultrafast characteristics of linear and nonlinear optical operations while circumventing electronic bottlenecks. The all-optical computer realizes linear operations, nonlinear functions, and memory entirely in the optical domain with accuracy surpassing a purely linear model up to 80 GHz clock rates depending on the task. We experimentally demonstrate a prototypical task of noisy waveform classification as well as perform ultrafast in-situ analysis of the soliton states from integrated optical microresonators. We further illustrate the application of the architecture for generative artificial intelligence based on quantum fluctuations to generate images even in the absence of input optical signals. Our results highlight the potential of all-optical computing beyond what can be achieved with digital electronics by utilizing ultrafast linear, nonlinear, and memory functions and quantum fluctuations.

## Abstract (web summary)

We demonstrate an ultrafast all-optical computer which greatly exceeds the limited clock rates of current electronic computers.

## Graphical abstract

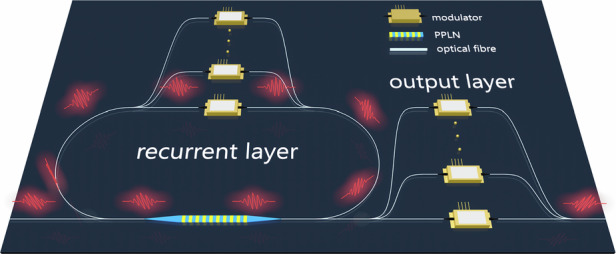

## Introduction

The clock rate ultimately determines the minimum time between sequential operations or instructions in a computer^1^ since computers cannot effectively process information or respond to input signals on timescales shorter than a single clock cycle. The evolution of computer hardware has been characterized by many major technological shifts: starting from early mechanical computers such as the Z1^2^ with a clock rate of 1 Hz, then progressing to general purpose electronic computers constructed from vacuum tubes such as ENIAC^3^ with a clock rate of 100 kHz, and finally maturing into today’s central processing units (CPUs)^1^ consisting of billions of integrated silicon transistors with GHz clock rates. Each dramatic increase in clock rate has historically enabled numerous new applications and innovations which were previously computationally infeasible.

**Fig. 1:**
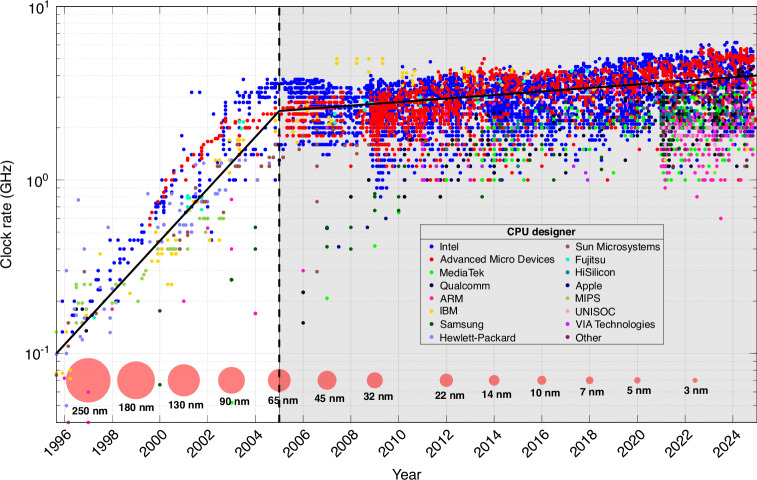
**CPU clock rates over the past 29 years**. Each point indicates the clock rate and testing date for a different type of commercially-available CPU. The color corresponds to the entity that designed the CPU, with some prominent CPU designers listed in the legend. Red circle and text bottom insets represent increasingly advanced semiconductor process nodes and approximately when they were introduced. Prior to 2005, clock rates increased exponentially and commensurately with Moore’s Law. However, clock rates have stagnated and only increased incrementally since 2005

Modern CPU clock rates have stagnated at ∼5 GHz since circa 2005 as shown in Fig. 1. Prior to 2005, CPU clock rates increased commensurately with Moore’s Law^4^. This abrupt change primarily results from the breakdown of Dennard scaling^5^ for transistors at the device level and the increasing prevalence of the von-Neumann bottleneck^6^ at the system level, which prompted CPU designers to abandon further significant increases in clock rate. As a result, recent performance improvements are largely attributable to the introduction of multi-core and other highly parallel computer architectures that increase the overall computational throughput. Although the clock rate is not a directly comparable measure of absolute computing speed between different families of computer processors since the instruction sets and operations during each clock cycle may differ, it remains clear that the limited clock rates of electronic computers prevent real-time processing or control of emerging ultrafast information systems at picosecond or faster timescales. This highlights a unique opportunity for optical computing, particularly where all the computational operations, i.e., linear and nonlinear functions, as well as the memory, are realized in the optical domain. Implementing such an all-optical computing platform in a scalable and programmable fashion has proven challenging especially because of the limitations with the nonlinear functions and all-optical memory^7^.

Optical computers have experienced a resurgence in recent years as application-specific hardware for both linear operations^8–10^ and nonlinear functions^11–13^ in deep learning^14–16^, neuromorphic computing^17–21^, and combinatorial optimization^22–24^ workloads. However, previous approaches mainly prioritized energy-efficient, high-throughput, or parallel processing and still depend on digital processors or optoelectronics for intermediate computational steps, hence were ultimately bottlenecked by electronic response times. Additionally, several of the previous all-optical approaches^22,25–29^ suffered from one or a combination of (i) lacking crucial operations such as nonlinearity and/or memory, (ii) lack of programmability, and (iii) utilization of slow nonlinearities.

An all-optical and programmable computer that can exceed the clock rates of current electronic computers is lacking. In this work, we propose and experimentally demonstrate all-optical computing at high clock rates beyond the limitations of electronic computers by combining ultrafast nonlinear optics for nonlinear operations, interference for linear operations, and active cavities for optical memories. Ultrafast nonlinear optics provides two unique advantages over previous approaches: (i) the near-instantaneous response time of the parametric ${\chi }^{(2)}$ optical nonlinearity is orders-of-magnitude faster than electronic nonlinearities, and (ii) the ability to generate ultrashort laser pulses, which allows for time-multiplexing and higher single-channel data-encoding rates compared to electronics or continuous-wave light. We provide several experimental examples of computing tasks including noisy waveform classification, in-situ processing of native ultrafast optical input signals, time-series forecasting, and all-optical image generation seeded from quantum noise.

## Results

### All-optical computer architecture

**Fig. 2:**
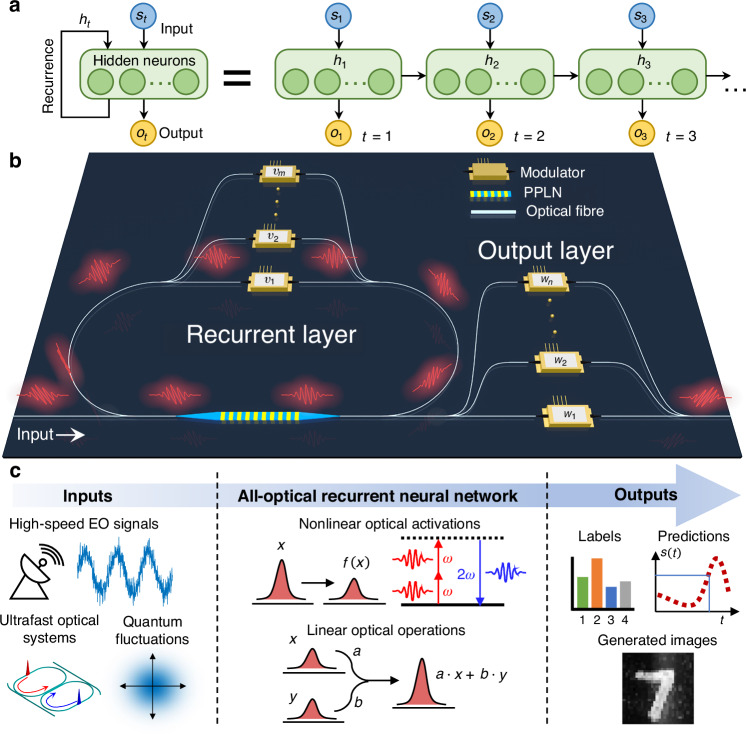
**All-optical computer architecture**. **a** A general recurrent neural network (RNN) consists of an input layer, hidden layer with recurrent connections, and output layer. **b** Schematic of the all-optical recurrent neural network (AO-RNN) architecture based on a time-multiplexed photonic network with (**c**) ultrafast optical inputs undergoing optical feedback and recurrent connections, nonlinear optical activations, and linear optical operations to produce optical outputs

Our optical computer architecture employs a recurrent neural network (RNN) framework as shown in Fig. 2a. An RNN contains an input layer, hidden recurrent layer, and output layer^30^. Unlike purely feed-forward architectures, this kind of driven dynamical system is well-suited for temporal or sequential information processing due to the inherent memory endowed by recurrent neuron states that are propagated between successive time steps. We also note that RNNs are Turing-complete^31^. Compared to digital or von-Neumann computer architectures, the RNN architecture lends itself more naturally to ultrafast optics since it is inherently analog and utilizes a dynamical memory instead of non-volatile memory elements. We construct an experimental proof-of-concept for an all-optical recurrent neural network (AO-RNN) using off-the-shelf optical fiber components, with operating wavelength of $\lambda \approx$ 1.55 µm, as shown in Fig. 2b. The AO-RNN is based on a time-multiplexed photonic network^22,32,33^ in which information and input data sequences $\left\{{s}_{t}\right\}$ are encoded onto the coherent amplitude of ultrashort laser pulses. Therefore, the effective clock rate of the AO-RNN is equivalent to the laser pulse repetition rate ${f}_{\,c}$. We utilized different kinds of optical frequency combs^34^ including mode-locked lasers, electro-optic frequency combs, and optical microcombs to generate input signals for different tasks. Recurrent connections between time steps are performed using an active optical cavity, which acts as an optical feedback loop. The optical cavity also contains intra-cavity linear couplings implemented using a multi-arm Mach-Zehnder interferometer in which the coupling weights are encoded using electro-optic amplitude modulators in each arm. The specific network topology of the recurrent layer is determined by the lengths of the optical delay lines in each arm, so the temporal delays ${T}_{m}$ should be an integer multiple of the clock period $1/{f}_{\,c}$. In-line nonlinear activation functions are performed using a reverse-proton exchange periodically-poled lithium niobate (PPLN) waveguide^35^. The PPLN enables strong ${\chi }^{(2)}$ nonlinear optical processes such as pump-depleted second-harmonic generation at low pulse energies, which results in a sigmoid-like input-output function for the pulse amplitude at the fundamental harmonic^36^. Finally, the linear output layer is performed using another multi-arm Mach-Zehnder interferometer with weights encoded by electro-optic modulators and connections determined by temporal delay lines. Therefore, the AO-RNN is a fully-analog optical computer that accepts ultrafast optical inputs and produces optical outputs through a combination of linear operations, nonlinear activations, and memory feedback entirely in the optical domain.

### Noisy waveform classification

**Fig. 3:**
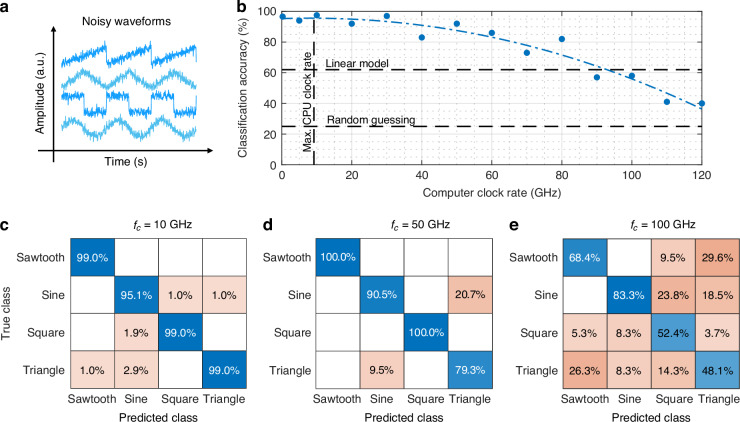
**All-optical noisy waveform classification**. **a** Temporal sequences of sawtooth, triangle, square, and sine waves corrupted with noise are classified by the AO-RNN. **b** The measured classification accuracy (blue dots) generally decreases as the clock rate ${f}_{\,c}$ increases. The bottom horizontal dashed line corresponds to random guessing accuracy of 25%, the upper horizontal dashed line corresponds to the accuracy of a purely linear model, and the vertical dashed line indicates the maximum achievable clock rates by digital electronic computers. Therefore, a nonlinear optical computational advantage is achieved in the top-right region. The (column-normalized) confusion matrices are shown for clock rates of **c** ${f}_{c}=$ 10 GHz, **d** ${f}_{\,c}=$ 50 GHz, and **e** ${f}_{c}=$ 100 GHz

To evaluate the maximum achievable clock rate ${f}_{\,c}$ of the experimental AO-RNN, we trained it (see Materials and Methods) to perform the prototypical task of noisy waveform classification. We consider four classes of temporal waveforms: sawtooth, triangle, square, and sine. The waveforms have the same period and duration, but are also corrupted by some random noise as shown in Fig. 3a. The waveform samples are fed sequentially as a single-channel input into the AO-RNN, and the task is to classify each waveform into the correct class. Therefore, this task is inherently single-threaded and cannot necessarily be accelerated through parallel processing. The AO-RNN produces a single-channel optical output pulse for each optical input pulse. To assign a single class label for verification purposes, the output pulses are photodetected, which effectively acts as a low-pass filter performing temporal global average pooling. The average optical output is compared against threshold decision boundaries (Materials and Methods) in digital postprocessing to assign a final class label. Alternatively, if multiple output channels are available, then a conventional softmax classification can also be applied. Notably, the requisite digital postprocessing for class label assignment occurs only after the entire duration of the waveform sequence, hence it only needs to be performed at a much lower rate compared to the clock rate ${f}_{\,c}$ of the AO-RNN and does not bottleneck the computation. In principle, the output optical pulses containing information about the classification result could be used as the input to a second optical computer stage for further optical processing if desired.

The classification accuracy of the AO-RNN generally decreases as the clock rate ${f}_{c}$ increases as shown in Fig. 3b. It achieves a peak classification accuracy of 97.5% at a clock rate of ${f}_{c}=\,$10 GHz (Fig. 3c), which exceeds the clock rate of commercially-available CPUs. The classification accuracy decreases to 92% at a clock rate of ${f}_{\,c}=$ 50 GHz (Fig. 3d) as the AO-RNN begins to confuse some sine/triangle waveforms. The classification accuracy further decreases to 58% at a clock rate of ${f}_{c}=$ 100 GHz (Fig. 3e) as the AO-RNN also confuses square/sine and triangle/sawtooth waveforms. Even at the maximum tested clock rate of ${f}_{\,c}=$ 120 GHz, the AO-RNN still achieves a classification accuracy significantly higher than random guessing. Repeating the same task using a purely linear model (i.e., without nonlinear activation function) with ${f}_{\,c}=$ 10 GHz achieves a classification accuracy of only 62%, which confirms that the PPLN optical nonlinearity is important for achieving a high classification accuracy. The accuracy of the AO-RNN outperforms the linear model accuracy at clock rates up to ${f}_{\,c}=$ 80 GHz. We designate an AO-RNN with both a clock rate higher than any electronic CPU and classification accuracy higher than a linear model as exhibiting a “*nonlinear optical advantage*” for high-clock rate computing applications.

We have identified several factors contributing to the decline in classification accuracy as the clock rate ${f}_{\,c}$ increases.

First, for testing purposes, we generated the input signals electro-optically using optical time-interleaving techniques (Supplementary Information Section V), which becomes increasingly more difficult as ${f}_{\,c}$ increases. The AO-RNN relies on coherent interference for performing linear operations and memory feedback, hence the relative phase and temporal separation of coherent laser pulses is critically important. Consequently, computational fidelity degrades with increasing phase noise and timing jitter in input signals. Second, although the ${\chi }^{(2)}$ optical nonlinearity is of the ultrafast variety and near-instantaneous, the PPLN possesses a finite phase-matching bandwidth ∼100 GHz. Therefore, the effectiveness of the nonlinear activation function, and hence classification accuracy, begins to degrade when ${f}_{\,c}$ exceeds the phase-matching bandwidth. Finally, neighboring pulses begin to overlap temporally and experience undesirable cross-talk when the clock period $1/{f}_{\,c}$ becomes comparable to the pulse duration.

### Native ultrafast optical signals

Many of these limitations can be circumvented when input data originates natively in the optical domain, thus not requiring electro-optic or optoelectronic conversions for generating input signals. Fortunately, the study of ultrafast optics is rife with examples of optical signals that possess a high degree of coherence and occur on timescales that are too fast for real-time processing or control by electronic computers. Here, we propose the AO-RNN as an analytical in-situ tool for ultrafast optics, which can potentially enable new functionalities that are infeasible using existing experimental techniques.

**Fig. 4:**
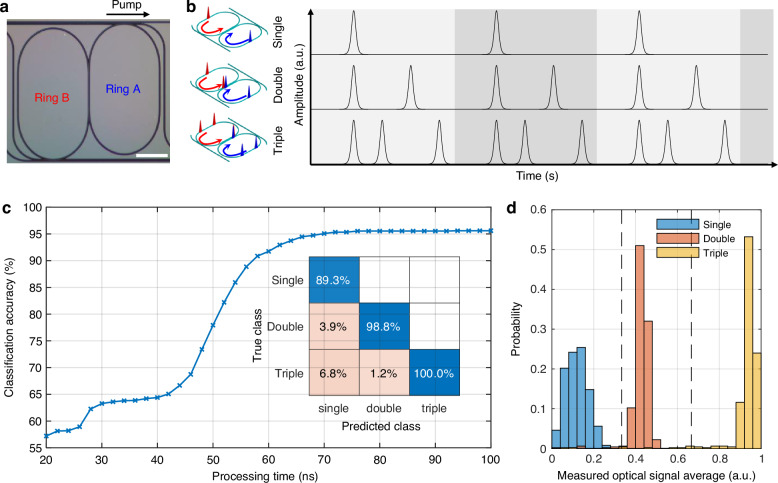
**Microcomb bipartite soliton state classification**. **a** Micrograph of a Si_3_N_4_ integrated coupled microresonator (white scale bar: 1 mm). **b** Bipartite bright soliton states with single, double, or triple pulse pairs. **c** Classification accuracy of soliton states with different AO-RNN processing times. Inset: confusion matrix for a processing time of 100 ns. **d** Histogram and decision boundaries for measured optical output averages with a processing time of 100 ns

For example, Fig. 4a shows ultra-low-loss Si_3_N_4_ integrated coupled microresonators^37^. This configuration can be operated as an optical frequency comb or “microcomb” through the balance of ${\chi }^{(3)}$ optical nonlinearity and dispersion, giving rise to bipartite bright-soliton states. Microcombs have attracted immense research interest due to their technological importance in a wide range of applications including optical computing, LIDAR, dual-comb spectroscopy, low-noise microwave synthesis, optical metrology, and astrocombs^9,10,38^. The Si_3_N_4_ microcomb can produce different soliton states including a single soliton pulse pair, double soliton pulse pair, or triple soliton pulse pair (Supplementary Information Fig. S6). The output optical signal in the time-domain is a periodic waveform composed of sub-picosecond pulses with repetition rate of ${f}_{\,c}\approx$ 19.97 GHz as shown in Fig. 4b. We consider the task of classifying bipartite soliton states (single, double, or triple) given that the optical waveforms have the same average power, repetition rate, center wavelength, polarization, and spectral bandwidth. This is challenging to do in real-time since the optical waveforms are too fast for direct photodetection. Moreover, conventional optical measurement techniques employing spectrometers or autocorrelation typically require scanning elements at millisecond or slower timescales. Other ultrafast characterization methods such as time-stretching^39–41^ struggle with the combination of both high-repetition rate and high-duty cycle, which is needed to distinguish the bipartite soliton states. Existing single-shot^42^ or few-shot^43^ methods are limited by low frame update rates and/or require extensive postprocessing algorithms to extract the useful information. Additionally, our all-optical computing architecture can be realized as a feedback mechanism for controlling the soliton state generation and/or ultrafast sensing schemes based on similar soliton dynamics^44^.

The AO-RNN can classify bipartite soliton states with varying amounts of processing time given by the length of the input optical waveform as shown in Fig. 4c. The final class label is assigned in the same way as for noisy waveform classification.

The AO-RNN achieves a high classification accuracy of 95.6% for processing times shorter than 100 ns. The minimum processing time or latency is limited by the main cavity roundtrip light propagation time in the AO-RNN, which is ∼24 ns. We observe that multiple cavity roundtrips are necessary to ensure high classification accuracy. This latency is a consequence of using relatively long optical-fiber components/connectors, and can be drastically reduced by direct splicing or instead using a corresponding integrated photonic platform with sub-nanosecond roundtrip times. Therefore, it is possible to use the AO-RNN as an in-situ tool for near-real-time and few-shot classification of optical soliton states, which can enable faster measurement and feedback control loops.

### Time-series forecasting

The previous two example tasks were classification tasks. We demonstrate that the AO-RNN can additionally perform regression tasks such as time-series forecasting with a high clock rate. Faster regression capabilities are essential for real-time decision-making in many applications including quantitative finance^45^, experimental particle physics^46^, and optical signal processing^47^. In this task, samples of a time-series are encoded onto the coherent amplitude of optical pulses and inputted one at a time into the AO-RNN. The task is to predict the next value in the time-series given the current input value and past inputted values. The corresponding output pulse should have an amplitude representing the predicted value of the next time step sample.

**Fig. 5:**
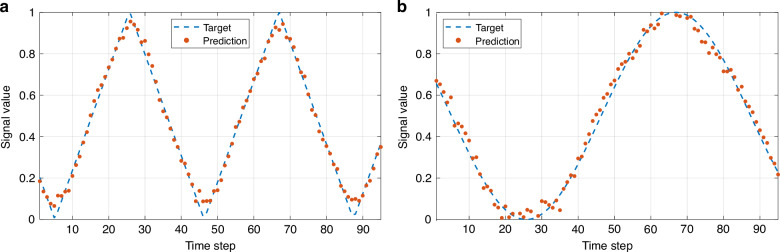
Temporal waveform prediction. Predicted (dots) one-step-ahead values for target (dashed lines) **a** triangle and **b** sine input time-series

We show two examples of time-series forecasting for triangle and sine waveforms in Fig. 5. The AO-RNN predictions show close agreement with the target values for these simple waveforms, and achieves normalized mean square error (NMSE) as low as 0.0144 and 0.0094, respectively, up to a clock rate of ${f}_{\,c}=$ 10 GHz. Unlike for classification tasks in which the speed of the output measurement can be amortized over the entire duration of the time-series, regression tasks require single-shot and rapid output measurements. In this case, our ability to test and verify the output predictions is limited by the maximum bandwidth of our photodetector that is ∼25 GHz. Although the AO-RNN can operate with far higher clock rates, as evidenced by the noisy waveform classification task, we were experimentally limited in accurately measuring the real-time pulse-to-pulse output values beyond a clock rate of ${f}_{\,c}=$ 10 GHz. This may be improved by using a faster photodetector up to ∼ 100 GHz and better data acquisition tools or optical time-division demultiplexing schemes^48^. Nevertheless, this is not a fundamental limitation of the AO-RNN, but rather a constraint of our current output measurement techniques. If an output remaining in the optical domain is sufficient, then the clock rate may be much higher since the optical outputs can in principle directly serve as optical inputs for another optical computer or actuator.

### Image generation seeded from quantum fluctuations

Finally, we demonstrate an example of a generative task in which the AO-RNN can use quantum fluctuations as the seed to generate images in the absence of any input optical signals^49,50^. The central problem for generative models is to learn a complicated unknown target distribution from which samples (e.g., images) are available, and then to use the model to efficiently generate new samples from the target distribution. We take inspiration from recent advances in generative artificial intelligence based on diffusion^51^ and flow-based^52^ models. In this case, a simple known distribution (typically a standard Gaussian distribution) is continuously perturbed to match the unknown target distribution. Then, the learned mapping can be applied to an initial random sample from the simple distribution to generate a new sample from the target distribution.

**Fig. 6:**
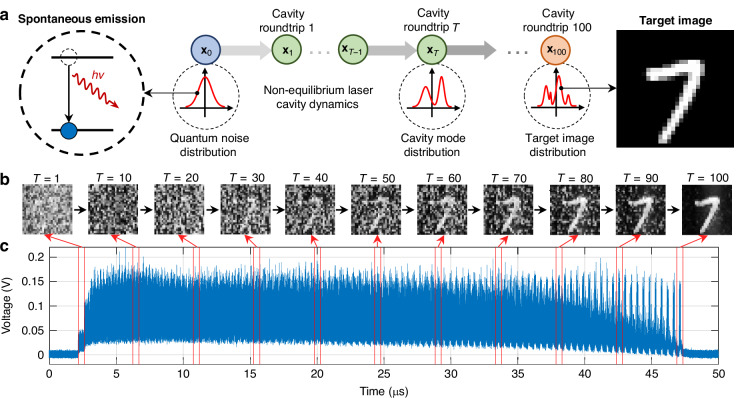
**Quantum all-optical image generation**. **a** An all-optical image generator maps a quantum noise distribution from spontaneous emission into an unknown target image distribution of MNIST handwritten digits using highly non-equilibrium laser cavity dynamics. **b** Example images after every 10 cavity roundtrips and **c** measured cavity output time trace up to *T* = 100 roundtrips representing a generated training sample of a seven

We trained the AO-RNN using the MNIST handwritten digits dataset^53^ to generate 28 × 28 greyscale images of the class “seven” as shown in Fig. 6a. This implementation utilizes only the AO-RNN’s recurrent layer. The main cavity contains an optical amplifier that acts as a programmable gain/loss mechanism. The optical amplifier also supplies quantum noise in the form of spontaneous emission, which approximately follows a Gaussian distribution (Supplementary Information Fig. S9).

Therefore, the AO-RNN can be interpreted as a highly nonlinear laser cavity. In conventional lasers, spontaneous emission in the gain medium serves as the spark to initiate stimulated emission, whereupon population inversion and gain-clamping after the laser threshold (i.e., gain equals loss) is exceeded will lead to a steady-state laser emission^54^. Here we modulate the gain and intra-cavity connection weights at a clock rate ${f}_{\,c}$ that is much faster compared to the cavity roundtrip time. Therefore, the lasing dynamics are also highly non-equilibrium. We define cavity modes by virtually splitting the cavity into equally-spaced time bins. Each time bin corresponds to a different pixel location in the image and the average power contained in each time bin encodes for the pixel greyscale intensity. Upon initially turning on, the AO-RNN starts from spontaneous emission in the optical amplifier and then gradually generates a macroscopic image after $T=$ 100 cavity roundtrips by effectively controlling which cavity modes temporarily go above/below lasing threshold.

An example time trace of the cavity dynamics and resultant image generated of a sample during the training process is shown in Fig. 6b, c. Examples of new images (i.e., not part of the original MNIST dataset) of sevens generated after the training process and seeded by quantum noise are shown in Supplementary Information Fig. S10. We note that our simple generative AO-RNN exhibits limited model expressiveness and struggled to generate high-quality images of multiple different digit classes or learn more complicated image distributions beyond the MNIST dataset. We believe that the quality and diversity of generated sevens from this simple proof-of-concept model is promising, and better model expressiveness can be achieved through incorporating more expressive nonlinearities^11^ or leveraging more sophisticated hybrid optical learning techniques^55^.

In addition, the clock rate for this task was limited by the 10 GHz bandwidth of the electro-optic modulators used to update weights for linear operations. Unlike previous tasks, it is necessary to use fast weight updates to achieve sufficient model expressiveness for image generation. Therefore, the current implementation of AO-RNN for the image generation task is strictly a hybrid electro-optic system. A truly all-optical method could potentially involve combining the AO-RNN concept with diffractive optical neural networks for linear operations with a larger number of weights^26^.

## Discussion

In the present implementation of the AO-RNN, the clock rate ${f}_{c}$ is equal to the repetition rate of the optical pulses in the time-multiplexed network. For simplicity, this should match the input data rate of the desired input signal, e.g., the input sampling rate for electro-optic signals or the intrinsic repetition rate for periodic ultrafast optical systems. Therefore, the maximum achievable clock rate of the AO-RNN is fundamentally limited by the speed of the nonlinear activation function since it acts in-line on every optical pulse. In this case, the speed of the PPLN for in-line nonlinear activations is determined by the phase-matching bandwidth for ${\chi }^{\left(2\right)}$ nonlinear optical processes. The analog electrical bandwidth of the modulators for implementing linear operation weights does not affect the computer clock rate ${f}_{\,c}$ for inference tasks using static weights. However, for better model expressiveness, the modulator weights can be updated in real-time, ideally matching the clock rate ${f}_{\,c}$.

We opted to use off-the-shelf optical fiber components for simplicity, however, this was not ideal for optimal computer performance. For example, the PPLN used for nonlinear activations based on a weakly-guiding reverse-proton exchange waveguide^35^ has a relatively small phase-matching bandwidth of ∼100 GHz. However, we previously demonstrated higher-performance nonlinear activation functions using thin-film lithium niobate (TFLN) with maximum allowable clock rates *>*13 THz^11^. Furthermore, other devices demonstrated in TFLN, such as high-speed electro-optic modulators with *>*100 GHz bandwidth^56^ or all-optical switches with speeds *>*10 THz^12^ can enable faster real-time updating of weights. Faster and fully programmable weight update speeds comparable to the clock rate would also improve the model expressiveness in all tasks performed. The longest duration laser pulse width used in our experiments for the noisy waveform classification and time-series prediction tasks with electro-optic input signals was ∼5 ps, which limits the maximum allowable clock rates since pulses will begin overlapping in time and suffering from undesirable cross-talk beyond ∼200 GHz clock rates. This issue can be overcome by using even shorter laser pulses, for example, few-cycle pulses generated using nonlinear optical pulse compression^57^ in TFLN. Finally, implementing the main cavity in the AO-RNN using an integrated optical parametric oscillator^58–60^ in TFLN could also drastically reduce the overall latency given by the light propagation time through the network.

Therefore, the AO-RNN could greatly benefit from on-chip integration using TFLN^61^, which provides a scalable path towards all-optical computing at terahertz clock rates.

The conventional definition of clock rate may not directly apply to some kinds of asynchronous or clock-less analog computers^15,62^. Here, we use the concept of clock period more generally to mean the minimum time between successive computer operations. This concept can thus still apply to clock-less processors since there is a characteristic physical timescale associated with each nonlinear device (electronic, optoelectronic, optical, or otherwise). The key advantage of the AO-RNN is that it implements all linear operations, nonlinear activations, and memory feedback directly in the optical domain so that all operations can simultaneously benefit from exceptionally high clock rates without an electronic bottleneck for some parts of the computation. In this work, we focused on gaining an advantage through the clock rate of all-optical computers performing single-threaded programs and tasks. This allows us to move towards a new regime of picosecond-timescale processing that is currently inaccessible using electronic computers. However, another important consideration is the overall computational throughput (total operations per second). During each clock period, the experimental AO-RNN currently performs a maximum of 8 operations (1 nonlinear activation and 7 multiply-accumulate operations). Therefore, the current AO-RNN implementation has a tradeoff between single-channel clock rate and overall computational throughput. Related work by Nakajima et al.^21^. (see Supplementary Information Section VII for comparison) using a photonic reservoir computer with time and wavelength division multiplexing demonstrated higher computational throughput of 44.16 TMAC/s. Future work can combine the high clock rates of all-optical computers with parallel photonic processing techniques^10,33^ to also achieve superior computational throughput compared to electronic computers.

We note that the AO-RNN only performs all-optical computing during inference with static weights. However, the training process (see Materials and Methods) used a feedback loop with a digital electronic computer to perform gradient descent. Thus, it is a hybrid opto-electronic system during training and only all-optical during inference. A completely end-to-end all-optical system during both training and inference may be achieved by using optical training methods^63^.

One important caveat is that we have primarily compared our results to conventional silicon-based CMOS computer chips. In the context of high-speed electronic computing, there has also been interest in using more advanced materials and fabrication methods^64^ or superconducting circuits^65,66^ operating up to ∼50 GHz clock rate. However, these approaches have yet to scale beyond simple small-scale programs and/or require cryogenic cooling. In contrast, our AO-RNN has demonstrated complex temporal information processing at high clock rates and room temperature. We believe that the most useful near-term applications for this kind of ultrafast optical computer will be those in which the input signal occurs natively in the optical domain, hence bypassing the need for electro-optic input signal generation. Some prime examples include in-situ analysis of ultrafast imaging and spectroscopy data^67,68^, optical signal processing for high-speed coherent telecommunications systems^47,48,69^, and precision ranging or LIDAR using femtosecond lasers^70,71^.

In conclusion, we have successfully leveraged ultrafast nonlinear optics to develop a novel kind of all-optical computer that can surpass the limited clock rates of existing digital electronic computers. The proof-of-concept experimental results demonstrate that the AO-RNN can operate effectively at clock rates between 10 and 80 GHz depending on the type of computational task performed, which is a large increase compared to the ∼5 GHz clock rates of modern commercially-available CPUs. This work highlights significant progress towards a new regime for ultrafast optical computing, enabling nascent applications requiring real-time information processing and feedback control at picosecond timescales.

## Materials and methods

### Experimental setup

A detailed schematic of the experimental setup for the AO-RNN is shown in Supplementary Information Fig. S1. Wherever possible, we used commercially-available, single-mode polarization-maintaining (PM) optical fiber components with center operating wavelength of $\lambda \approx$ 1.55 µm. The recurrent layer consists of a main cavity and contains an intra-cavity Mach-Zehnder interferometer with two arms. The relative temporal delay ${T}_{0}$ between the arms of the Mach-Zehnder interferometer determines the connection topology, and is a hyper-parameter of the AO-RNN to be chosen depending on the task. For proper operation, ${T}_{0}$ is chosen to be an integer multiple of the clock period $1/{f}_{\,c}$. The relative delay is fine-tuned using a homemade free-space delay stage since it is difficult to cut/splice optical fibers to precisely match the desired length. The approximate temporal delay is set by propagating a single reference laser pulse and manually moving the stage. Then, fine-tuning is done by a high-precision linear micrometer stage to maximize the pulse temporal overlap by observing the coherent interference fringe visibility. The weights are set using electro-optic amplitude modulators (IXBlue MXAN-LN-10) in each arm of the Mach-Zehnder interferometer. Each modulator accepts a constant bias voltage input to set the operating point, and also a high-speed RF voltage input to rapidly modulate the amplitude of optical signals. Bias voltages are controlled using an ADC/DAC (National Instruments 782258) and RF waveforms up to 12 GSa*/*s are generated using an arbitrary waveform generator (Keysight M8190A). The voltages are the learnable weight parameters of the AO-RNN. We only trained the bias voltages for the noisy waveform classification, optical soliton classification, and time-series forecasting tasks. We set the bias point to “closed” for the image generation task and trained the RF voltages at a 10 GSa*/*s update rate. The main cavity additionally contains a fiber-coupled PPLN waveguide^35^ with a wavelength-division multiplexer on both the input/output to separate the fundamental harmonic ($\lambda \approx$ 1.55 µm) and second harmonic ($\lambda \approx$ 0.775 µm). The second harmonic signal is only used to monitor the phase-matching of the PPLN, and is not used as part of the AO-RNN computation. The phase-matching of the PPLN is adjusted using a heater stage and thermocouple controller (Omega CSC32), with an optimal operating temperature around ∼51.5 ^◦^C. The maximum average optical power in the main cavity must be *<*100 mW to avoid photo-induced damage to the PPLN. The PPLN was bypassed for the noisy waveform classification task using a purely linear model. A booster optical amplifier (Thorlabs S9FC1004P) was used to set the overall gain/loss in the main cavity for the image generation task. The amplifier was bypassed for the noisy waveform classification, optical soliton classification, and time-series forecasting tasks since we desired to have a fading memory property for these tasks. The main cavity roundtrip light propagation time was ∼24 ns for the noisy waveform classification, optical soliton classification, and time-series forecasting tasks. The main cavity roundtrip light propagation time was ∼453 ns for the image generation task since we required more time bins for a sufficient number of pixels to generate MNIST images. For the image generation task, the output of the recurrent layer was directly detected since the output layer is not used. For the other tasks requiring an output layer, the optical output of the recurrent layer is amplified using an erbium-doped fiber amplifier (Thorlabs EDFA100S) and filtered through a 200 GHz band-pass filter to minimize the amplified spontaneous emission noise before entering the output layer. The output layer consists of a four-arm Mach-Zehnder interferometer of similar construction as the one in the recurrent layer. Three arms encode the output layer weights, and the fourth arm is used as an optional constant optical bias. The relative delays, ${T}_{1}\approx$ 0.3 ns and ${T}_{2}\approx$ 1.6 ns, are hyper-parameters of the AO-RNN to be chosen depending on the task. Only the constant bias voltages were trained for the output layer modulators due to the limited number of available high-speed arbitrary waveform generator channels. Optical outputs are detected using a high-speed photodetector (Newport 1414) and stored on a real-time oscilloscope (Keysight MSOV334A) for post-processing if necessary. The high-speed RF and optical inputs are synchronized using a low-noise 10 MHz reference clock. The relative delay between generated RF and measured optical signal is calibrated using the sample marker output from the arbitrary waveform generator. We use a backwards-propagating locking reference, which is tapped from the unmodulated input laser source, to perform active phase-stabilization of the temporal delay lines. The recurrent layer and output layer each contain independent backwards-propagating optical locking loops based on a Pound-Drever-Hall scheme^72^. Slow photodetctors (Newport 2053) are used to measure the backwards-propagating locking signals. The electronic locking signals are input to proportional-integral derivative controllers (Red Pitaya STEMlab 125-14) and outputs are amplified (Thorlabs MDT693B) to drive fiber phase-shifters (General Photonics FPS-002-L) that actively stabilize the relative phase in each optical delay line.

### Input signals

We used a variety of different optical frequency combs to generate optical input signals for the experimental tasks. External optical signals input to the AO-RNN are gated using an electro-optic amplitude modulator, which is biased closed so that signals only enter the AO-RNN when desired to mark the start of a computation. For noisy waveform classification, the input waveforms were generated electro-optically using a high-speed arbitrary waveform generator and electro-optic amplitude modulators. The laser source for clock rates ${f}_{\,c}\in$ [250 MHz,5 GHz] was a fiber mode-locked laser (MenloSystems FC1500-250-WG) and the laser source for clock rates ${f}_{c}\in$ [10 GHz,120 GHz] was a homemade electro-optic frequency comb (Supplementary Information Section III). We can perform real-time input generation up to clock rates of ${f}_{c}\approx$ 48 GHz using optical time-interleaving (Supplementary Information Section V), and offline (i.e., prepared ahead-of-time) input generation up to clock rates of ${f}_{\,c}\approx$ 200 GHz using an asynchronously-pumped cavity. Each noisy waveform (sawtooth, triangle, square, and sine) was 120 periods in duration with a total of 5120 equally-spaced samples. The period for all noisy waveform classes was the same, and sawtooth/square waves had a duty cycle of 1*/*2. The ideal noiseless waveforms had normalized amplitudes in the range [−1,1] and the measured optical noise for the waveforms is approximately given by an additive Gaussian distribution with zero mean and standard deviation of ∼0.158 as shown in Supplementary Information Fig. S2. The Si_3_N_4_ coupled optical microresonators used to generate bipartite soliton states is of the same design as in ref. ^37^. We maintained the average input optical power to the AO-RNN at ∼5.3 mW for all bipartite-soliton states by monitoring on a slow thermal power meter (Thorlabs PM20). We carefully characterized the single, double, and triple pulse pairs by separately measuring the optical spectrum (Yokogawa AQ6370C), autocorrelation (Femtochrome FR-103XL), and RF beat-note (Rhode & Schwarz FSW85) of each state as shown in Supplementary Information Fig. S4. The input signals used for the time-series forecasting task were also generated electro-optically in the same way as for the noisy waveform classification. The triangle wave had 44 samples per period with a duty cycle of 1*/*2. The sine wave had the same sample time-spacing as the triangle wave but double the period.

### RNN model

The general RNN architecture is given by Eq. 1:

1a $${h}_{i}\left(t+1\right)={f}_{i}\left(\mathop{\sum }\limits_{j=1}^{N}{W}_{{ij}}^{r}\cdot {h}_{j}\left(t\right)+\mathop{\sum }\limits_{j=1}^{m}{W}_{{ij}}^{{in}}\cdot {s}_{j}\left(t\right)\right)$$

1b $${o}_{k}\left(t\right)=\mathop{\sum }\limits_{l=1}^{N}{W}_{{kl}}^{{out}}\cdot {h}_{l}\left(t\right)$$

where $t{\mathbb{\in }}{\mathbb{N}}$ is the discrete-time step (one time-step represents one clock period), ${\bf{h}}\in {{\mathbb{R}}}^{N}$ is the *N*-dimensional hidden recurrent layer activation, ${\bf{s}}\in {{\mathbb{R}}}^{m}$ is the *m*-dimensional input sequence, ${\bf{o}}\in {{\mathbb{R}}}^{n}$ is the *n*-dimensional output value, ${{\rm{W}}}^{{\rm{r}}}\in {{\mathbb{R}}}^{N\times N}$ is the matrix of recurrent layer weights, ${{\rm{W}}}^{{\rm{in}}}\in {{\mathbb{R}}}^{N\times m}$ is the matrix of input layer weights, ${{\rm{W}}}^{{\rm{out}}}\in {{\mathbb{R}}}^{n\times N}$ is the matrix of output layer weights, and ${f}_{i}{\mathbb{:}}{\mathbb{R}}{\mathbb{\to }}{\mathbb{R}}$ is an element-wise activation function for $i=1,\,2,\,...,N$ and $k=1,\,2,\,...\,,{n}$. We give a simplified model of the AO-RNN, which is similar to the rotating neuron architecture proposed in ref. ^73^, by dividing the main cavity into equally-spaced time bins containing pulses. The hidden recurrent layer has a cyclic structure with weights:

2 $${W}_{{ij}}^{r}=\left\{\begin{array}{c}{\alpha }_{i}\left(t\right),{\rm{if}}i-j\equiv 1\,\left(\mathrm{mod}\,N\right)\\ {\beta }_{j}\left(t\right),{\rm{if}}i={i}_{r}\,{\rm{and}}\,j\in {T}^{r}\\ 0,{\rm{otherwise}}\end{array}\right.$$

where $\left\{{\alpha }_{i}\left(t\right)\right\}$ are weights representing the loss/gain factor for the pulse propagating from time bin $i$ to time bin $({i}+\,1)\,\mathrm{mod}{N}$. The weights $\left\{{\beta }_{j}\left(t\right)\right\}$ represent the intra-cavity couplings between time bin ${i}_{r}$ and other time bins in ${T}^{r}$. The set ${T}^{r}$ of time bin indices represents the choice of optical delay lines in the intra-cavity Mach-Zehnder interferometer. In our experimental AO-RNN, ${T}^{r}=\left\{{j}_{r}\right\}$ represents a single connection since we used a two-arm Mach-Zehnder interferometer. This cyclic structure is a special case of the fully-connected RNN model and is still Turing-complete, however, in practice may lead to reduced model expressiveness for some tasks. We used constant weights for the noisy waveform classification, optical soliton classification, and time-series forecasting tasks, and time-varying weights for the image generation task. Our AO-RNN has single-channel input/outputs, ${m}={n}=\,1$, due to experimental constraints. In this case, the input weights are given by ${{\rm{W}}}^{{\rm{in}}}=\varepsilon {{\bf{e}}}_{{i}_{0}}$ where ${{\bf{e}}}_{k}$ is the $k$^th^ unit vector, ${i}_{0}$ is the index of the time bin coupled to the input line, and $\varepsilon$ is the input coupling factor. We treat the input scaling $\varepsilon$ as a hyper-parameter that is not trained. However, it is also possible to employ more complicated input masking techniques, such as in previous time-multiplexed photonic reservoir computers^27,28^. The output weights are given by ${W}_{1l}^{{out}}={\gamma }_{l}\left(t\right)$ if $l\in {T}^{{out}}$, and 0 otherwise, where $\left\{{\gamma }_{l}\left(t\right)\right\}$ are determined by the output layer modulators and ${T}^{{out}}$ is the set of time bin indices representing the choice of optical delay lines in the output layer Mach-Zehnder interferometer. For the noisy waveform and optical soliton classification tasks, we additionally perform temporal global average pooling of the optical power to yield a single output value $y={\left\langle {\left|o\left(t\right)\right|}^{2}\right\rangle }_{0 < t\le L}$, over the entire input sequence of length $L$, and normalize the output averages $\left\{y\right\}$ to the range [0,1]. The predicted class label is assigned by comparing $y$ against threshold decision boundaries ${{\mathbb{Z}}}_{q}/q$ where $q$ is the number of classes, such that $y$ belongs to class $p\in {{\mathbb{Z}}}_{q}$ if $\left(y\ge p/q\right)\wedge \left(y < (p+1)/q\right)$. Alternatively, the more conventional softmax classification can be used if the number of output channels in the AO-RNN equals the number of classes. For the image generation task, only the recurrent layer is used. The input sequence can be replaced by an additive noise term (Supplementary Information Fig. S9), representing the amplified spontaneous emission from the optical amplifier, and the output samples are then equivalent to sampling from a single point in the cavity. Strictly speaking, the nonlinear laser cavity becomes a continuous-field distribution so the concept of discrete time bins is not well-defined. However, we discretize the cavity field based on the fast output measurement sampling time $\tau$. Then, during each cavity roundtrip, we assign the value ${p}_{v}$ for pixel $v\in {{\mathbb{Z}}}_{V}$ in an image sequence with $V$ pixels by coarse-graining the fast output samples into slower time bins with period ${t}^{{\prime} }$ such that ${p}_{v}={\left\langle {\left|o\left(\tau \right)\right|}^{2}\right\rangle }_{v{t}^{{\prime} }\le \tau < \left(v+1\right){t}^{{\prime} }}$. Pixel values are rescaled to be in the range [−1,1] for each cavity roundtrip. We used a time bin period of ${t}^{{\prime} }=$ 0.4 ns and applied symmetric zero-padding for unused pixels in each roundtrip (∼453 ns) since MNIST images only contain 784 pixels.

### Training procedure

We use a model-agnostic forward-only training algorithm based on the method proposed in ref. ^16^. For each training iteration, we perform the following steps:

1. Choose a random direction vector $\Delta \in {\left\{+\delta ,-\delta \right\}}^{d}$ where $d$ is the number of trainable model parameters, the elements of $\Delta$ are sampled from a Bernoulli distribution ${\Delta }_{i} \sim B(1/2)$ for $i=1,\,2,\,...,d$, and $\delta$ is the step size.
2. Perturb the model parameters $\Theta \in {{\mathbb{R}}}^{d}$ by ∆ and perform a forward-pass through the model to evaluate the loss function ${\mathcal{L}}{\mathscr{(}}\Theta +\Delta )$.
3. Perturb the model parameters $\Theta$ in the opposite direction $-\Delta$ and perform a forward-pass through the model to evaluate the loss function ${\mathcal{L}}{\mathscr{(}}\Theta -\Delta )$.
4. Estimate the directional derivative of the loss as:
  3 $${\nabla }_{\Delta }{\mathcal{L}}\left(\Theta \right)\approx \frac{{\mathcal{L}}\left(\Theta +\Delta \right){\mathscr{-}}{\mathcal{L}}\left(\Theta -\Delta \right)}{2\mathrm{||}\Delta \mathrm{||}}$$
5. Update the model parameters: $\Theta \to \Theta -\eta {\nabla }_{\Delta }{\mathcal{L}}\left(\Theta \right)\Delta$ where $\eta$ is the learning rate.

For the experimental AO-RNN, the forward-pass steps are performed directly in the optical hardware, but the other training steps are performed on a digital computer. During testing, the trained parameters are frozen and so the forward-pass inference is all-optical. The model parameters in our experimental AO-RNN correspond to electro-optic modulator voltages (both DC and RF). The half-wave voltages are ${V}_{\pi }\approx$ 6 V and we found that a perturbation step-size of $\delta \,=$ 0.02 V with learning rate of $\eta \,=$ 2 × 10^−3^ was adequate for all our experimental tasks. For the noisy waveform and optical soliton classification tasks, we used a mean squared error loss ${\mathcal{L}}=\left\langle {\left(y-z\right)}^{2}\right\rangle$ where $z=(2p+1)/(2q)$ is the midpoint of the decision boundaries for the true class label. For the time-series forecasting task, we used a mean squared error loss ${\mathscr{L}}={\left\langle {\left[o\left(t\right)-s\left(t+1\right)\right]}^{2}\right\rangle }_{t}$ for the one-step-ahead prediction. For each training sample in the image generation task, we used a diffusion process to generate intermediate target values during each cavity roundtrip $T$:

4 $${{\bf{z}}}_{T-1}=\sqrt{1-{\sigma }_{T}}\cdot {{\bf{z}}}_{T}+\sqrt{{\sigma }_{T}}\cdot \epsilon$$

where $\epsilon \sim {\mathscr{N}}\left(0,{\bf{I}}\right)$ is standard Gaussian noise, ${{\bf{z}}}_{100}$ is the ideal target from the image dataset, and ${\sigma }_{T}$ is a noise-variance schedule that increases linearly each from ${\sigma }_{100}=\,$0.001 to ${\sigma }_{1}=$ 0.02. We used a mean-squared error ${\mathscr{L}}={\left\langle {\left[{{\bf{x}}}_{T}-{{\bf{z}}}_{T}\right]}^{2}\right\rangle }_{{t}^{{\prime} },T}$ taken over all roundtrips and time bins for each sample where ${{\bf{x}}}_{T}$ are the measured pixel values for each roundtrip $T$. The training and testing sample sizes for each task are shown in Table 1.

**Table 1:** Sample sizes for experimental AO-RNN tasks

Task	Batch size	Iterations	Testing size
Noisy waveform classification	4	200	800
Optical soliton classification	10	150	1500
Time-series forecasting	460	200	9200
All-optical image generation	12	1200	-

### CPU clock rates

Each scatter point in Fig. 1 represents the clock rate and testing date for a different commericially-available CPU. The data was collected from a variety of online CPU benchmarking sources^74,75^ and press/product release information from prominent CPU designers. We consider CPUs with the same architecture design but different generations, performance tiers, or optimizations as different processors. For each processor, we show the maximum reported clock rate, including possible turbo clock rates. For processors containing multiple cores operating at different clock rates, we show the clock rate of the fastest core. We restrict our attention to general purpose CPUs designed for desktops, laptops, servers, tablets, smartphones, wearable devices, etc; however, we exclude application-specific hardware accelerators such as graphics or tensor processing units, which typically have far lower clock rates compared to CPUs. The maximum CPU clock rate used for the dashed vertical line in Fig. 2b is based on the current (as of 14 March 2024) CPU-Z overclocking world-record of 9117.75 MHz^76^.

## Supplementary information

Supplementary information (final)

## Data Availability

The data used to generate the plots and results in this paper are available from the corresponding author upon reasonable request.

## Code availability

The code used to analyze the data and generate the plots for this paper is available from the corresponding author upon reasonable request.
