# Supplementary material for: All-optical computing towards 100-GHz clock rates
Source: Light Sci Appl. 2026 Jul 17;15:321. doi: 10.1038/s41377-026-02314-5 (PMC13379394; doi:10.1038/s41377-026-02314-5)
Supplement: Supplementary file 1 — Supplementary information (final) [file 41377_2026_2314_MOESM1_ESM.pdf]

Supplementary Information for "All-optical computing towards 100-GHz clock rates"

Gordon H.Y. Li[1,*], Midya Parto[2,3,4,*], Jinhao Ge[5,*], Qing-Xin Ji[5], Maodong Gao[3,5], Yan Yu[5], James Williams[2], Robert M. Gray[2], Christian R. Leefmans[1], Nicolas Englebert[2], Kerry J. Vahala[5], and Alireza Marandi[1,2,†]

[1]Department of Applied Physics, California Institute of Technology, Pasadena, CA 91125, USA

[2]Department of Electrical Engineering, California Institute of Technology, Pasadena, CA 91125, USA

[3]Physics and Informatics Laboratories, NTT Research, Inc., Sunnyvale, California 94085, USA

[4]CREOL, The College of Optics and Photonics, University of Central Florida, Orlando, FL, USA

[5]T. J. Watson Laboratory of Applied Physics, California Institute of Technology, Pasadena, California 91125, USA

[*]These authors contributed equally

[†]marandi@caltech.edu

I. EXPERIMENTAL SETUP

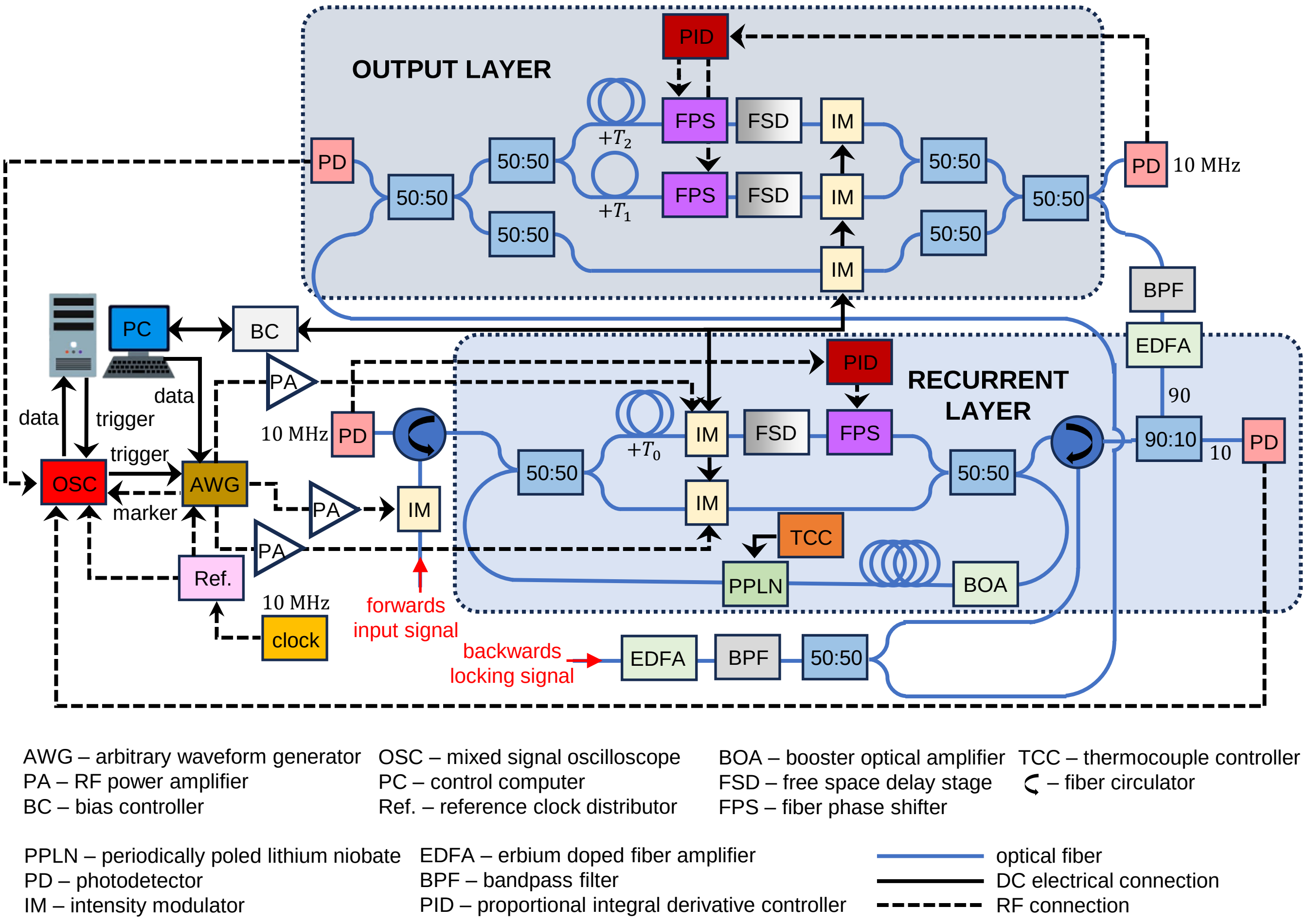

FIG. S1. **Detailed schematic of AO-RNN experimental setup.**

II. NOISY WAVEFORMS

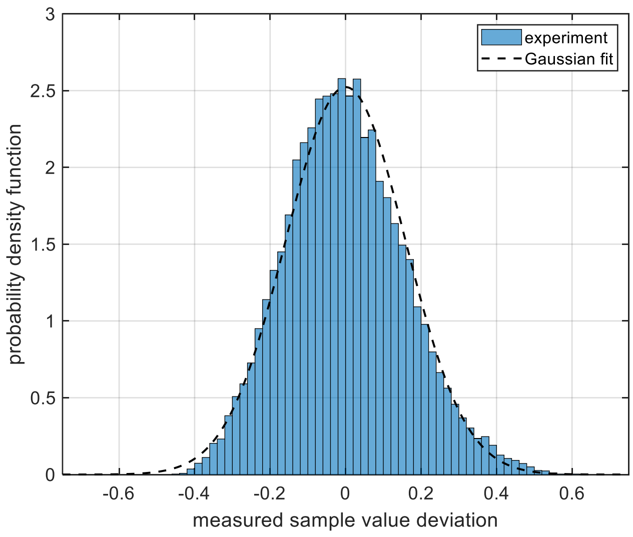

FIG. S2. **Noise distribution for noisy waveform input signals.** The histogram shows the statistics for the deviation between the ideal noiseless waveform samples and the measured optical input signal values (amplitudes normalized to [-1,1]) for 8000 input waveform sample points. It is well-approximated by a Gaussian distribution (dashed black line) with zero mean and standard deviation of ~ 0.158.

III. ELECTRO-OPTIC FREQUENCY COMB

The physical configuration of the electro-optic frequency comb system is illustrated in Fig. S3. A continuous-wave, single-frequency laser is phase-modulated by three cascaded modulators, generating a series of sidebands that form

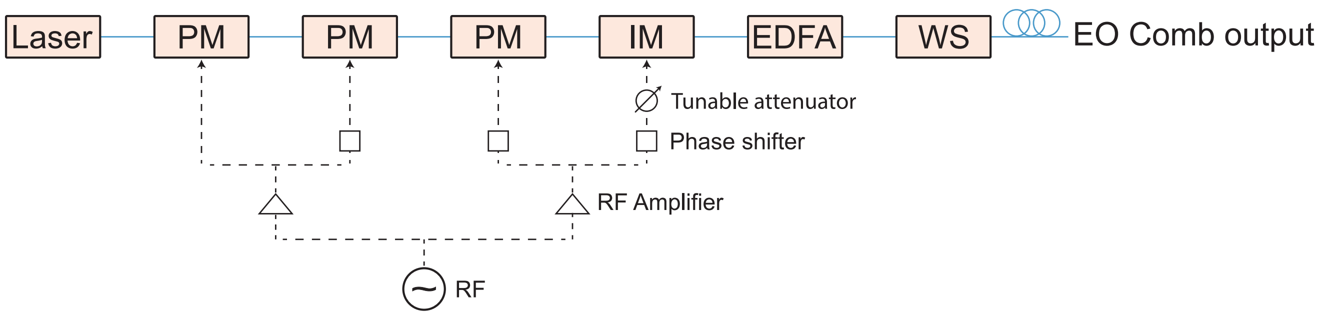

FIG. S3. **Experimental setup for Electro-optic frequency comb.** Abbreviations: PM, phase modulator; IM, intensity modulator; EDFA, erbium-doped fiber amplifier; WS, waveshaper.

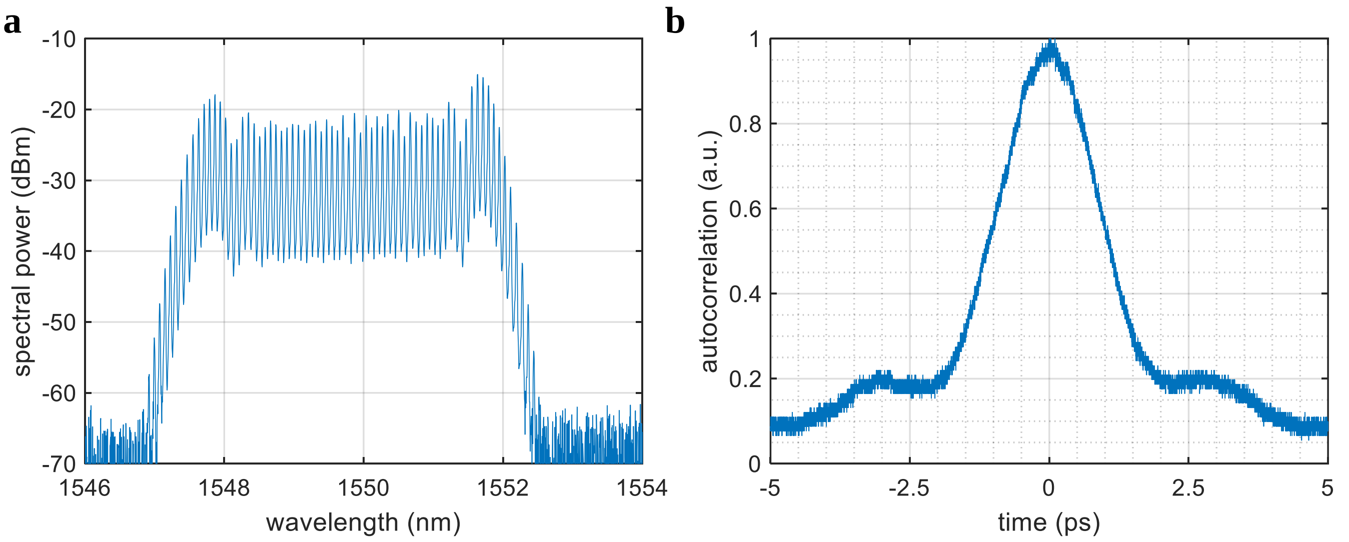

FIG. S4. **Electro-optic frequency comb characterization.** (a) Optical spectrum and (b) autocorrelation for the electro-optic frequency comb operating with a repetition rate of 10 GHz.

a frequency comb, as shown in Fig. S4(a). The spacing between adjacent comb lines is determined by the radio-frequency (RF) signal driving the phase modulators, which is set to 10 GHz in our experiment. To maximize the number of comb lines, the RF signals driving the individual modulators are amplified and phase-shifted to ensure effective in-phase modulation. Following phase modulation, an additional intensity modulation stage is introduced to flatten the comb spectrum. The intensity modulator (IM) is biased at the half-power point of its transmission curve, and the modulation signal frequency matches that of the phase modulators. The modulation strength is controlled using a variable RF attenuator. Subsequently, the comb is amplified using an erbium-doped fiber amplifier (EDFA) to facilitate further operations with enough power. To generate optical pulses in the time domain, the phase of each comb line is adjusted using a programmable waveshaper, which applies second-order dispersion compensation to the frequency comb. After phase compensation, the time-domain field becomes a series of optical pulses, characterized by an autocorrelator, as shown in Fig. S4(b). The dispersion applied by the waveshaper is optimized by minimizing the pulse width measured through autocorrelation.

IV. SOLITON MICROCOMB

The coupled-ring device is fabricated using an ultra-low-loss Si_3N_4 platform [1]. It consists of two partially coupled racetrack resonators with slightly different free-spectral ranges (FSRs), which provide anomalous dispersion required

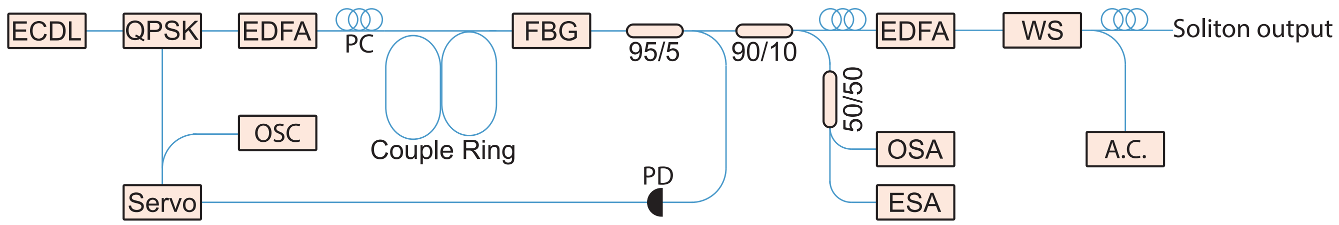

FIG. S5. **Experimental setup for generating the multi-soliton state in coupled-ring resonators.** Abbreviations: ECDL, external cavity diode laser; QPSK, quadrature phase shift keying; EDFA, erbium-doped fiber amplifier; FBG, fiber Bragg grating; PC, polarization controller; PD, photodetector; OSA, optical spectrum analyzer; ESA, electrical spectrum analyzer; WS, waveshaper; OSC, oscilloscope; A.C., autocorrelator.

for bright soliton generation. The laser output is modulated by a fast single-sideband modulator (QPSK) and subsequently amplified by an erbium-doped fiber amplifier (EDFA). The QPSK modulator facilitates the rapid frequency sweeping of the pump laser in order to avoid thermal effect during the soliton locking process [2]. Lens fibers (not shown in Fig. S5) are used to couple light in and out of the coupled-ring system. The through-port output is filtered using a fiber Bragg grating (FBG) to separate the comb and pump signals. The filtered signal is then split into multiple beams for different purposes. One beam is directed to a photodetector, which measures the comb power that are used for stabilizing the pump-resonance detuning. Another beam is sent to an optical spectrum analyzer (OSA) and an electrical spectrum analyzer (ESA) for characterizing soliton spectrum and RF beat-note signal. The remaining beam, carrying the main comb power, is amplified by an EDFA and then passed through a waveshaper to compensate the fiber dispersion. The amplified soliton is then directed to the AO-RNN (all-optical recurrent neural network) setup.

When single, double, or triple soliton pulse pairs are formed in the resonator, the measured total comb power varies accordingly. By selectively stabilizing specific comb power levels, different stable soliton states can be reliably and individually locked for classification experiments, as shown in Fig. S6. For comparative analysis, the multi-soliton states are further characterized and validated using optical spectrum measurements and autocorrelation signals. These characterization results provide a detailed basis for the classification experiments conducted with the AO-RNN setup. The soliton spectrum and autocorrelation traces for different pulse numbers are shown in Fig. S6. The soliton spectrum exhibits an approximately $sech^2$ envelope shape, with dispersive waves [3] appearing at frequencies where the mode and comb frequencies coincide, leading to resonant power enhancement. The soliton repetition rate, measured using the ESA, is approximately 19.97 GHz with a resolution bandwidth of 1 kHz. The autocorrelation of the generated periodic soliton pulse train is measured using an autocorrelator, and the result is shown in Fig. S6. For single, double,

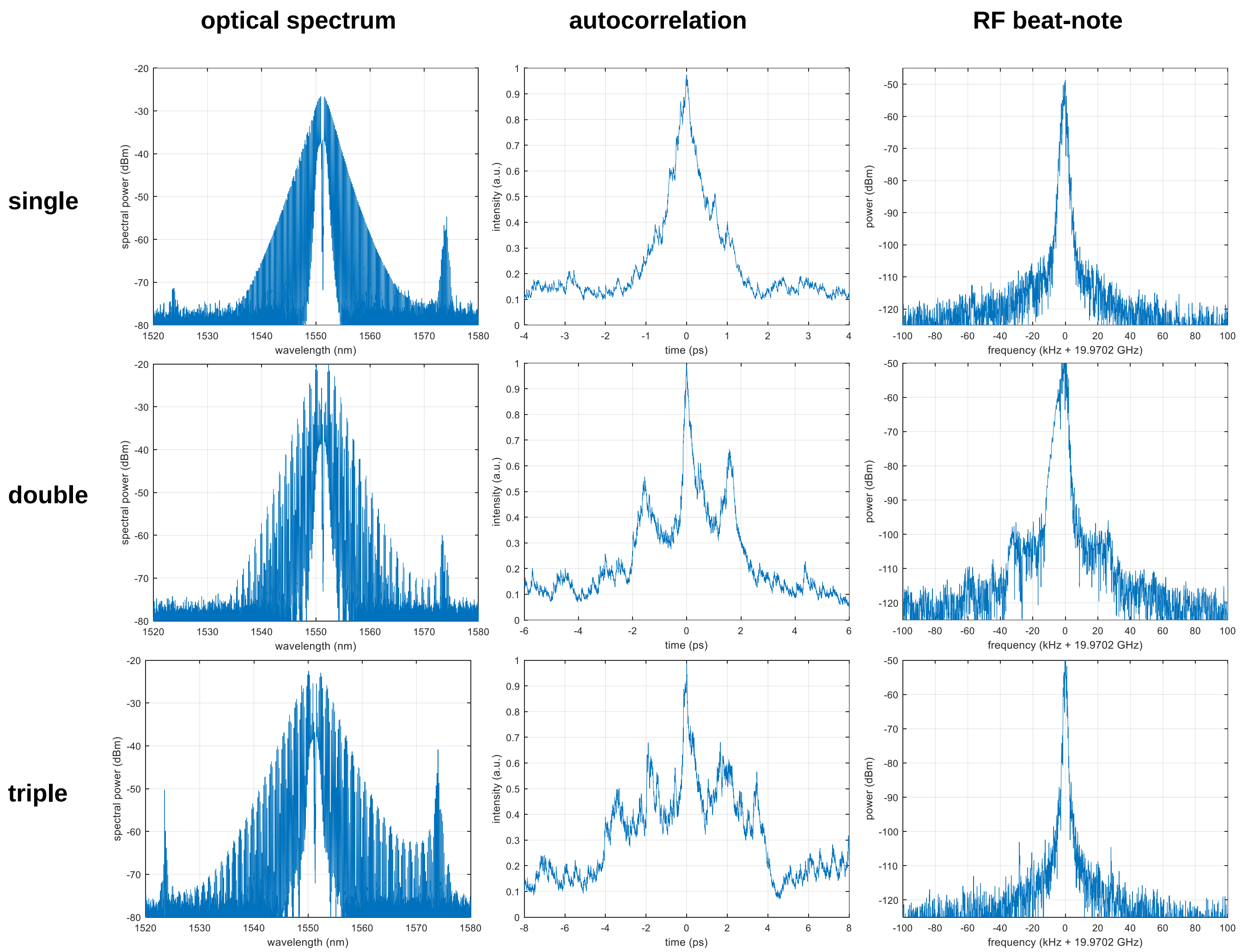

FIG. S6. **Bipartite-soliton pulse pair state characterization.** The optical spectrum (left column), autocorrelation (middle column), and RF beat-note (right column) for the single (top row), double (middle row), and triple (bottom row) bipartite-soliton pulse states.

and triple soliton states, the autocorrelation traces display 1, 3, and 5 peaks, respectively. As an additional note, the formation location of solitons within the resonator is a random process and is not actively controlled during the experiment (only the number of solitons is controlled). The data presented in Fig. S6 are selected as representative results.

V. ULTRAFAST OPTICAL INPUT TIME-MULTIPLEXING

The signal information in the AO-RNN is encoded onto the coherent amplitude of ultrashort laser pulses. One unique advantage of this approach over using continuous-wave light is that it allows for optical time-interleaving techniques to multiply the effective repetition rate and input sampling rate. For the task of noisy waveform classification, we can use two different time-multiplexing techniques to generate equivalent ultrafast optical input signals beyond the limited sampling rate of our arbitrary waveform generator. The first technique allows for real-time input generation as shown conceptually in Fig. S7. Consider an input laser pulse train with repetition period T. Suppose we wish to increase the effective input sampling rate by a factor of 4. Then, to do this, we use a Mach-Zehnder interferometer with 4 arms. Each arm is delayed by $T/4$ relative to the previous arm and contains an intensity modulator with sampling period of T. Upon recombining at the output, the optical input signal effectively has an input sampling period of $T/4$. This method exploits the fact that the pulse length of the laser pulse τ is much less than the RF sampling period T. The maximum allowable number of arms in the Mach-Zehnder interferometer to upconvert the

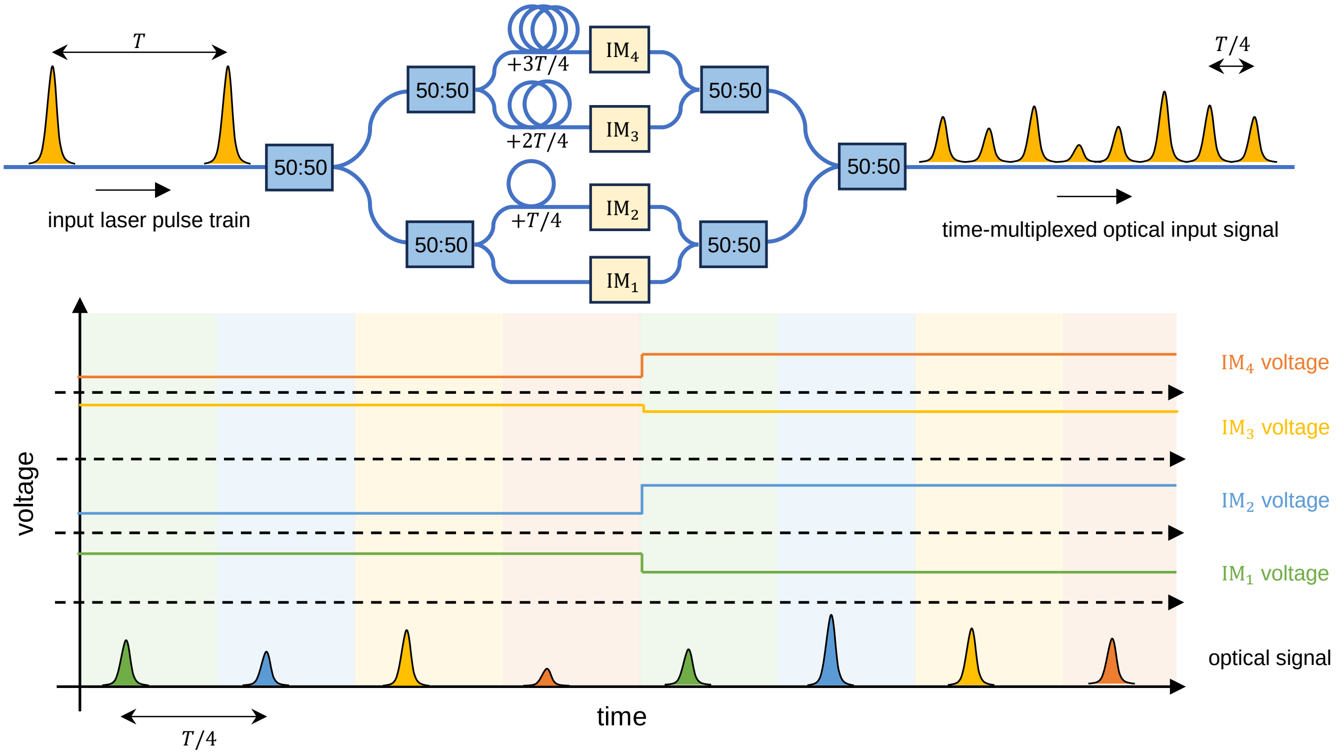

FIG. S7. **Real-time time-multiplexed optical input generation.** A multi-arm Mach-Zehnder interferometer in which each arm is delayed by an integer fraction of T relative to the other arms and contains an intensity modulator (IM) with sampling period of T can generate an optical input signal with effective sampling period that is an integer fraction of T.

sampling rate is $\sim T/\tau$ before the output optical pulses begin to undesirably overlap temporally.

In practice, we are limited by the number of available modulators and channels on our arbitrary waveform generator. The scope of this work is to demonstrate ultrafast optical computing, and not to design new ultrafast optical transceivers. Therefore, to reach even higher effective input sampling rates for very high clock rate computing, we use another time-multiplexing technique based on an asynchronously-pumped optical cavity. As opposed to real-time input generation, we call this technique "offline" input generation since the input signals must be prepared ahead-of-time before beginning the AO-RNN computation. The concept for offline input generation is shown in Fig. S8. Suppose that we have an input laser pulse train with repetition period T and we can generate RF samples with period T, but desire to have an input optical signal with sample period of T/m where $m > 1$. A synchronously-pumped optical cavity has a roundtrip time NT where N is an integer so that each input laser pulse will overlap temporally with a laser pulse in the cavity. We detune the cavity by adjusting a free-space delay stage such that the roundtrip time is reduced by T/m, so that the rountrip time of the asynchronously-pumped optical cavity is $NT - T/m$. During the first cavity roundtrip we modulate the input laser pulses with every m^{th} waveform sample (i.e. sample 1, $m+1$, $2m+1$, ...). Then, during the next cavity roundtrip we modulate the input laser pulses with every m^{th} waveform sample offset by 1 sample (i.e. sample 2, $m+2$, $2m+2$, ...). The cavity pulses from the first roundtrip will be displaced forwards by T/m relative to the input laser pulses for the second roundtrip. We repeat this procedure until we have completed modulation of all waveform samples. In this way, we gradually build-up the desired optical waveform with sampling period T/m over multiple cavity roundtrips using only a single input modulator and RF channel with sampling period T. The cavity contains an EDFA that is tuned to compensate the roundtrip loss so that many (typically > 20) cavity roundtrips are possible without exponential signal degradation from the cavity out-coupling and propagation loss. Amplitude scaling factors to account for loss variations between roundtrips can be calibrated by sending a single laser pulse into the cavity and measuring its amplitude decay after each roundtrip. Using this offline input generation method, the cavity roundtrip time must be at least $> nT/m$ where n is the total number of waveform samples. The maximum upconversion rate factor is limited by $\tau \approx T/m$ where τ is the input laser pulse length. If the cavity detuning T/m is comparable to the pulse length τ, then pulses will overlap from roundtrip to roundtrip, which will result in undesirable sample cross-talk. The gating modulator controlling inputs into the AO-RNN is synchronized to the offline input generation such that it only allows the final desired cavity roundtrip to be transmitted, which prevents input signal artifacts from the previous roundtrips.

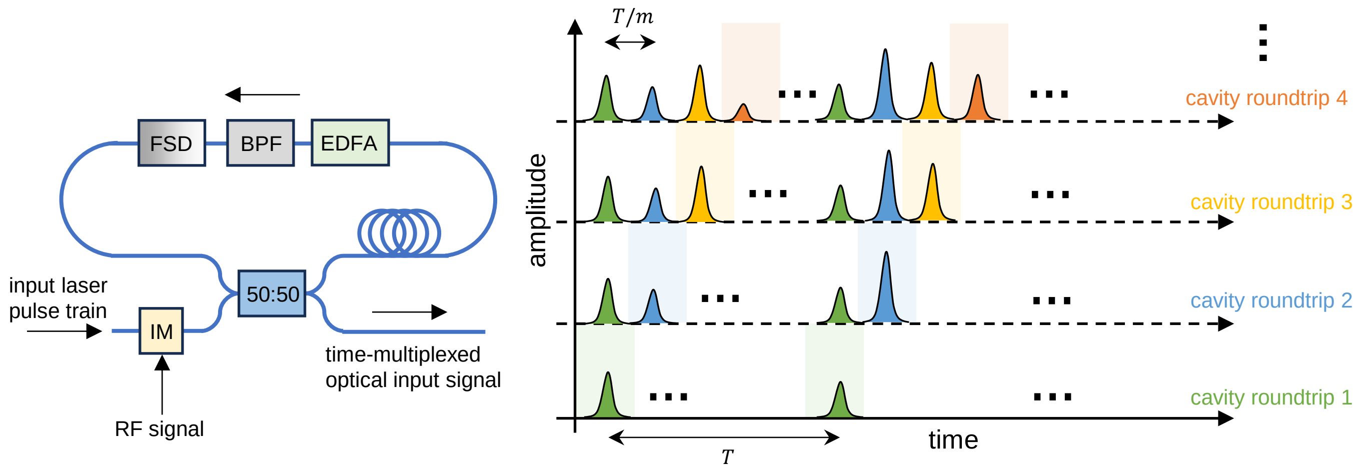

FIG. S8. **Offline time-multiplexed optical input generation.** The desired optical input signal is gradually built-up over many roundtrips of an asynchronously-pumped optical cavity. IM: intensity modulator, EDFA: erbium doped fibre amplifier, BPF: band-pass filter, FSD: free-space delay.

VI. ALL-OPTICAL IMAGE GENERATION

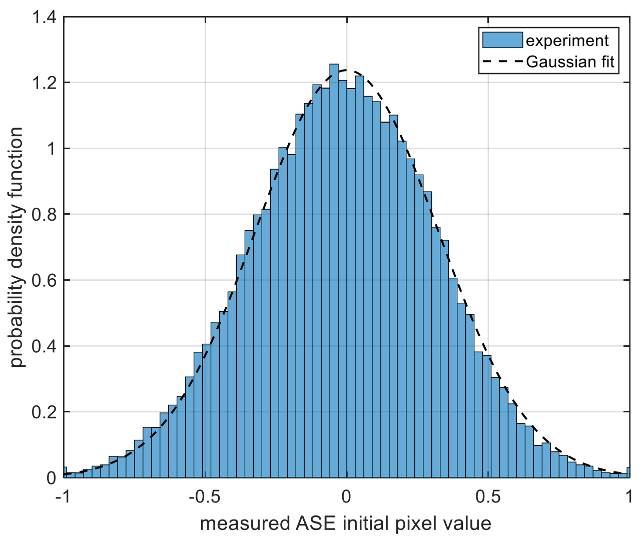

FIG. S9. **Quantum noise distribution.** The histogram shows the statistics for the initial pixel values sampled from amplified spontaneous emission (ASE) for ~ 35000 sample points. It is well-approximated by a Gaussian distribution (dashed black line) with zero mean and standard deviation of ~ 0.322.

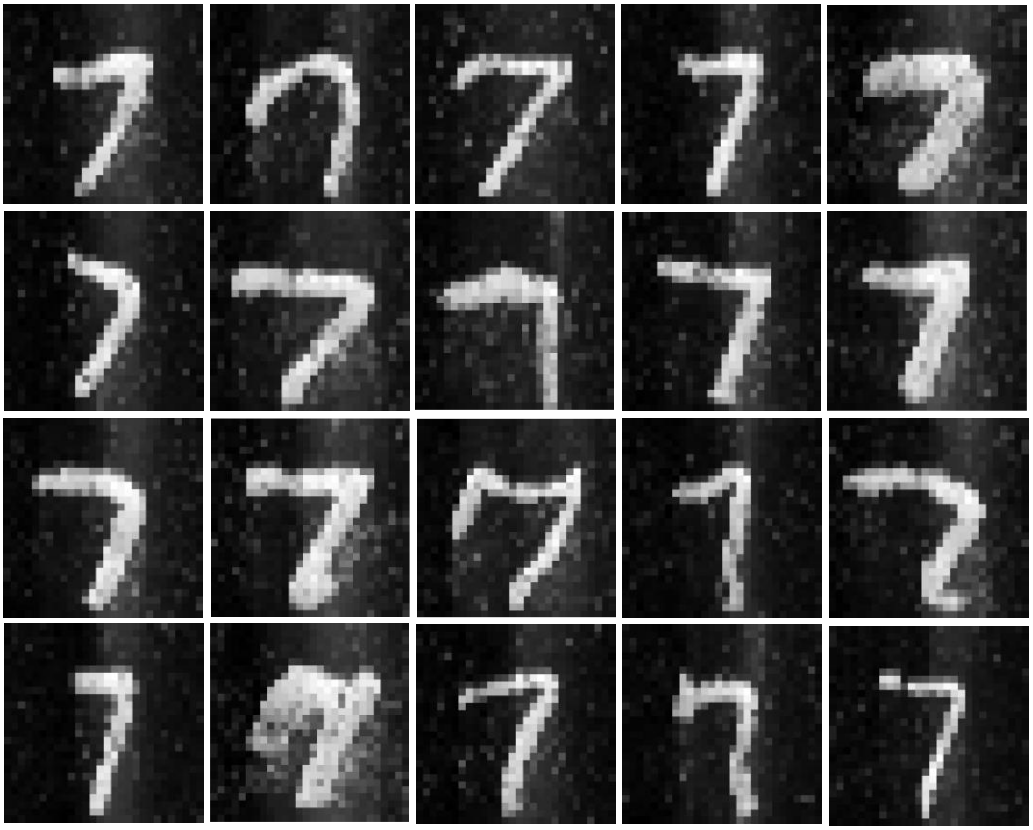

FIG. S10. **25 generated images of "seven" using the AO-RNN.**

VII. COMPARISON TO RELATED WORK

In this section, we discuss similarities and differences between Nakajima *et al.* [4] and this work. Both works utilize high optical bandwidths, temporal delay lines, and recurrent neural network architectures for various computational tasks. Essential features of the computing signals, operations, and metrics are compared in Table. I. The key difference is that Nakajima *et al.* [4] focus on maximizing the parallelism or computational efficiency to achieve an impressive proposed throughput as high as 1.77 PMAC$\cdot$s^{-1}. However, compared to the available optical bandwidth of $\sim$ 5 THz, the effective computational clock rate of $\sim$ 60 GHz is ultimately bottle-necked due to the use of RF input signals, electro-optics for nonlinear activations, and optoelectronics for the output layer. In contrast, this work takes advantage of an all-optical approach to achieve far higher computational clock rates in order to unlock a new regime of ultrafast information processing at picosecond timescales. This work utilizes optical input signals, performs all essential computational operations in the optical domain, and produces optical output signals. Therefore, this work is not limited by the same electro-optic/optoelectronic bottleneck as Nakajima *et al.* [4], however, we achieve this single-channel clock rate speed-up at the expense of a comparatively lower total throughput.

**Ref.**	**Nakajima *et al.*** [4]		**This Work**	
**version**	experimentally measured	proposed limits	experimentally measured	proposed limits
**platform**	planar lightwave circuit	planar lightwave circuit	optical fiber	thin-film lithium niobate
**linear ops.**	optical	optical	optical	optical
**nonlinear acts.**	electro-optic	electro-optic	optical	optical
**input signal**	electrical	electrical	optical	optical
**input layer**	optical	optical	optical	optical
**recurrent layer**	optical	optical	optical	optical
**output layer**	optoelectronic	optoelectronic	optical	optical
**output signal**	electrical	electrical	optical	optical
**wavelengths**	2	40	1	1
**pulse width**	30 ps	30 ps	5 ps	75 fs
**clock rate**	60 GHz	60 GHz	120 GHz	13.3 THz
**optical bandwidth**	5 THz	5 THz	200 GHz	13.3 THz
**throughput**	44.16 TMAC$\cdot$s^{-1}	1.77 PMAC$\cdot$s^{-1}	0.96 TOPS$\cdot$s^{-1}	106.7 TOPS$\cdot$s^{-1}

TABLE I. Comparison of optical computing signals, operations, and metrics between Nakajima *et al.* [4] and this work.

[1] W. Jin, Q.-F. Yang, L. Chang, B. Shen, H. Wang, M. A. Leal, L. Wu, M. Gao, A. Feshali, M. Paniccia, *et al.*, Hertz-linewidth semiconductor lasers using cmos-ready ultra-high-q microresonators, Nature Photonics **15**, 346 (2021).

[2] X. Yi, Q.-F. Yang, K. Y. Yang, M.-G. Suh, and K. Vahala, Soliton frequency comb at microwave rates in a high-q silica microresonator, Optica **2**, 1078 (2015).

[3] Q.-F. Yang, X. Yi, K. Y. Yang, and K. Vahala, Spatial-mode-interaction-induced dispersive waves and their active tuning in microresonators, Optica **3**, 1132 (2016).

[4] M. Nakajima, K. Tanaka, and T. Hashimoto, Scalable reservoir computing on coherent linear photonic processor, Communications Physics **4**, 20 (2021).
